# Super-resolution optical DNA Mapping via DNA methyltransferase-directed click chemistry

**DOI:** 10.1093/nar/gkt1406

**Published:** 2014-01-21

**Authors:** Charlotte Vranken, Jochem Deen, Lieve Dirix, Tim Stakenborg, Wim Dehaen, Volker Leen, Johan Hofkens, Robert K. Neely

**Affiliations:** ^1^Department of Chemistry, KU Leuven, Celestijnenlaan 200F, 3001 Heverlee, Belgium, ^2^Life Science Technologies, Imec, Kapeldreef 75, 3001 Heverlee, Belgium and ^3^Department of Chemistry, University of Copenhagen, Universitetsparken 5, 2100 Copenhagen, Denmark

## Abstract

We demonstrate an approach to optical DNA mapping, which enables near single-molecule characterization of whole bacteriophage genomes. Our approach uses a DNA methyltransferase enzyme to target labelling to specific sites and copper-catalysed azide-alkyne cycloaddition to couple a fluorophore to the DNA. We achieve a labelling efficiency of ∼70% with an average labelling density approaching one site every 500 bp. Such labelling density bridges the gap between the output of a typical DNA sequencing experiment and the long-range information derived from traditional optical DNA mapping. We lay the foundations for a wider-scale adoption of DNA mapping by screening 11 methyltransferases for their ability to direct sequence-specific DNA transalkylation; the first step of the DNA labelling process and by optimizing reaction conditions for fluorophore coupling via a click reaction. Three of 11 enzymes transalkylate DNA with the cofactor we tested (a readily prepared s-adenosyl-l-methionine analogue).

## INTRODUCTION

The direct visualization of specific sites on DNA molecules of many hundreds of kilobases in length can provide valuable genomic information, which often complements that derived from sequencing. For example, fluorescence *in situ* hybridization is often used to study structural variations within whole genomes (1). Optical mapping (2) uses enzymes to modify specific target sites of a few bases in length and images of these ‘tagged’ DNA molecules, up to megabases in length, can give a unique overview of genome structure. Optical mapping has found application in the scaffolding of sequence assemblies (3), the study structural variations (4) and strain-typing of organisms (5). Optical mapping is fundamentally a single-molecule approach, yet (similar to DNA sequencing) it typically necessitates the mapping of tens of overlapping DNA molecules to acquire a statistically sound map. This has a significant impact on the throughput of the experiment and, as a result, high ‘labelling’ efficiencies and fidelity are critical. Despite this, mapping of larger, e.g. human, genomes is possible (6), though pioneering work in this direction necessitated many weeks of continuous imaging (7). Technologies such as nanochannel-based DNA mapping (8) promise to increase the throughput of mapping experiments and have recently produced some spectacular results (9,10). However, a significant limitation of both optical (restriction enzyme-based) mapping and the nanochannel-based approach is that they result in maps with rather low density, typically one site per 10–20 kb. Hence, to use such maps to aid or validate *de novo* sequence assemblies, the sequencing data must be of extremely high quality. This results from the fact that reliable alignment of any given contiguous sequence assembly (contig) to the optical map requires that there are multiple map sites in that contig. Hence, contigs of >100 kb in length are typically required. There is a pressing need for the development of a mapping technology that bridges this gap, which has the density and accuracy to enable the assembly and validation of short contigs, on the scale of a few kilobases to tens of kilobases in length, such as those typically derived from a single sequencing experiment.

DNA mapping is a fundamentally single-molecule technology that can provide information on huge DNA molecules (up to megabases in length). It requires no DNA amplification or complex library preparation and, hence, in principle, it can be a quick and simple route to study whole genomes, even in complex samples. To this end, we aimed to develop an approach to mapping that would allow rapid single-molecule characterization of genomes using the DNA methyltransferases (of which there are many thousands known (11) to direct labelling.

We previously reported (12) the mapping of a bacteriophage genome (bacteriophage lambda), using DNA methyltransferase-directed transfer of activated groups (mTAG) (13–15), a labelling technology that uses a two-step labelling approach with a sequence specificity defined by a methyltransferase enzyme. We combined this with super-resolution localization microscopy to produce a map where the location of each site was determined to within 80 bp of its predicted site on the genome. However, our approach suffered from a significant limitation, in that we were only able to localize fluorophores at around a third of the targeted sites on any given DNA molecule. This was the result of a combination of factors, but mainly due to the low efficiency of the coupling chemistry (amino-to-NHS ester coupling) used. While we were able to assemble a complete map of the bacteriophage lambda genome using this approach, 20-fold coverage of the genome was needed to produce a reliable ‘consensus’ map. To extend our approach both to larger genomes and to more complex, non-uniform genomic samples (e.g. from environmental samples) we sought to drive this necessity to sample the genome multiple times over down.

**Figure 1.**
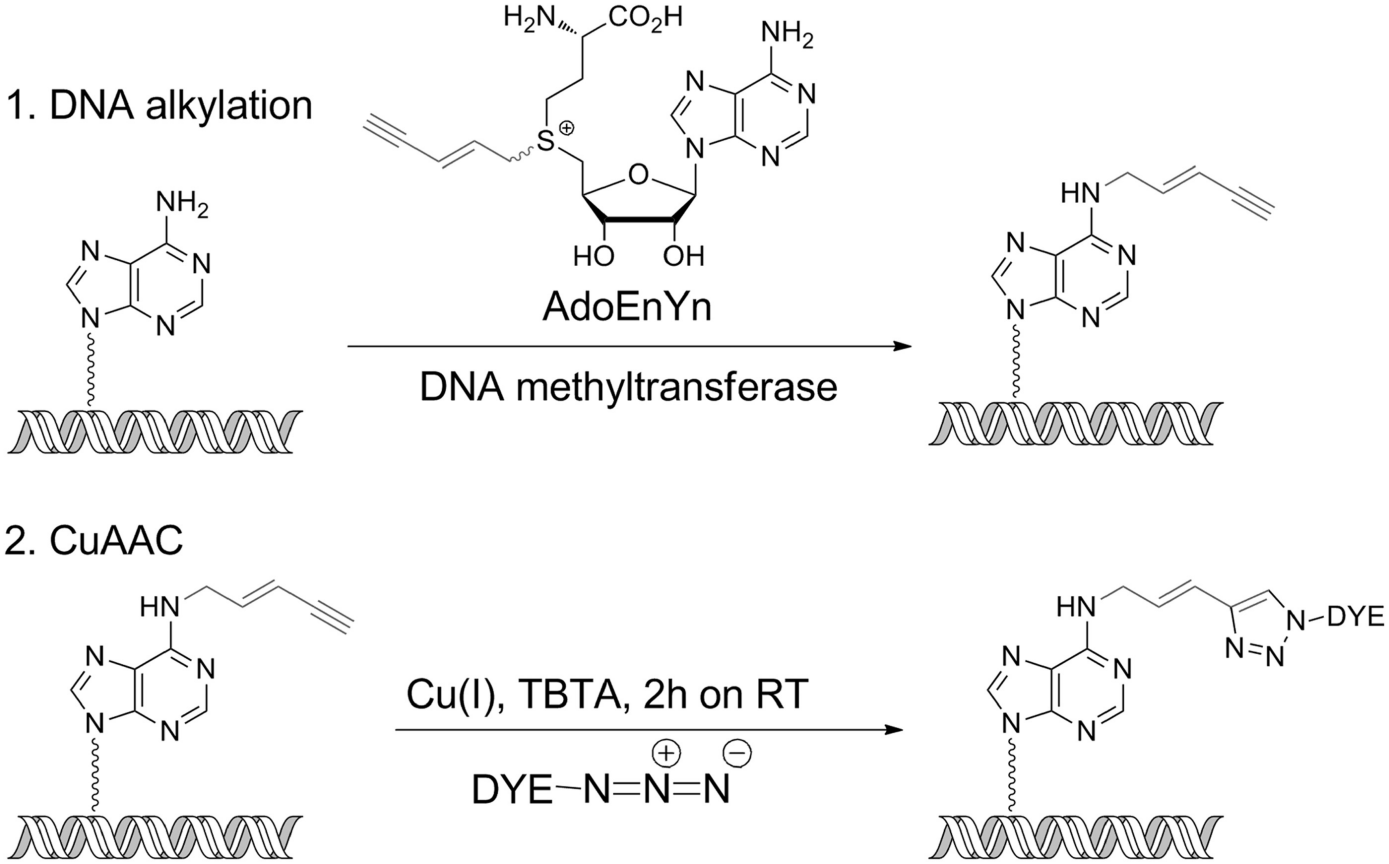
Methyltransferase-directed alkylation of DNA is followed by CuAAC at the alkylated site. (1) DNA is incubated along with a DNA methyltransferase enzyme (targeting adenine N6 for modification here) and a synthetically prepared cofactor (AdoEnYn). The methyltransferase is able to catalyse the transfer of the extended alkyl chain (grey) to the DNA duplex. (2) These modified sites on the DNA are subsequently fluorescently labelled via the CuAAC click reaction.

In fact, an ideal route to circumvent this problem would be to use the supreme efficiency of the DNA methyltransferase enzymes to directly couple a fluorophore to the DNA. This approach has been achieved previously using fluorescently modified aziridine-based cofactors (16,17). However, DNA modification in this case is stoichiometric, not catalytic, and most significantly only a limited number of enzymes display activity with any given cofactor (R.K.N., unpublished results). Hence, here we also use the two-step mTAG labelling approach but using an AdoMet analogue with a transferable alkyne moiety, which can be conjugated to a fluorophore using the highly efficient azide-alkyne (Huisgen) cycloaddition reaction. This approach has been successfully used for protein (18), DNA (19–22) and RNA labelling (23) using methyltransferase enzymes and we now extend its application to DNA mapping. The procedure uses the two steps summarized in Figure 1.

Here, we show that, of the 11 methyltransferases screened, 3 are capable of DNA transalkylation using 5′-[(S)-[(3S)-3-amino-3-carboxylpropyl](E)-pent-2-en-4-ynylsulfonio]-5′-deoxyadenosine, the AdoEnYn cofactor. Each has a distinct recognition sequence allowing enzymatic targeting of labels to 4-, 5- or 6-base DNA sequences. Coupling efficiencies of a fluorophore-azide (Atto647N) to these transalkylated DNA sites, using the copper-catalysed azide-alkyne cycloaddition (CuAAC) reaction are typically ∼70%. Hence, a true single-molecule map of the DNA sequence, with an average map density of approximately one site every 500 bases can be derived.

## MATERIALS AND METHODS

FokI methyltransferase was a kind gift from Bill Jack, NEB Inc. Clones for the M.BsaHI, M.XbaI, M.PvuII and M.PstI enzymes were a kind gift from New England Biolabs Inc. These enzymes were expressed and isolated as described previously (24). Briefly, the methyltransferase genes were inserted into the pTXB1 vector (NEB) (NheI and EcoRI sites). T7 Express competent cells (NEB) were transformed with these plasmids and, following induction with isopropyl ‘beta'-D-1-thiogalactopyranoside (IPTG), the cells were grown overnight at 30°C. The next day, cells were centrifuged, washed with phosphate buffered saline (Sigma) and lysed using a sonicator. Expressed methylases were purified using gravity-fed Ni-columns (Affiland). After incubating the cell lysate with the Ni-bead slurry, samples were washed at least five times with buffer containing 10 mM imidazole and eluted in different subfractions using 250 mM imidazole. Purity of the samples was checked using sodium dodecyl sulphate-polyacrylamide gel electrophoresis precast gels (NuPage 4–12% Bis–Tris gel, Invitrogen) that were stained with GelCode Blue (Pierce) before drying. Activity of the methylases was confirmed by incubation of the purified enzymes (0.1 mg/ml) with s-adenosyl-l-methionine (80 µM) and a suitable DNA (1 µg) substrate for 2 h. The DNA was subsequently challenged using the appropriate restriction enzyme and finally, this DNA was analysed using agarose gel electrophoresis (see Supplementary Data). Other enzymes were purchased from New England Biolabs Inc. unless otherwise stated.

T7 DNA was purchased from YorkBio. In a typical alkylation reaction, 1 μg of DNA was incubated for 4 h at 60°C in 20 µλ (1× NEB4 buffer) containing 2.5 μl of TaqI and 100 μM AdoEnYn cofactor. Subsequently, 1 μl of proteinase K was added to the reaction to digest the methylase enzyme. The DNA from this solution was ethanol precipitated and resuspended in 20 µl of phosphate buffered saline, pH 7.4. DNA labelling was carried out by incubating 10 μl of the alkylated DNA solution (∼0.5 µg of DNA) along with 0.5 µl of a 20 mM solution of Atto647N-azide [dissolved in dimethyl sulfoxide (DMSO)]. This was mixed with a solution containing 200 µM CuSO_4_, 5 mM sodium ascorbate and either 200 µM TBTA {tris[(1-benzyl-1*H*-1,2,3-triazol-4-yl)methyl]amine} or 2 mM THPTA {tris[(1-hydroxypropyl-1H-1,2,3-triazol-4-yl)methyl]amine}. The reaction was allowed to proceed for 2 h at room temperature.

The DNA was purified using an Illustra S-400-HR column (GE Healthcare). To further remove the free dye from the solution this was followed by ethanol precipitation of the DNA, incorporating multiple washing steps of the pelleted DNA. After resuspension, the DNA was used for deposition on glass coverslides (Fisher Scientific). The slides were cleaned extensively before use by sonication in acetone, 1 M NaOH (×2) and water. Slides were modified by pretreatment with an alkyl-silane (Aldrich).

The AdoEnYn cofactor was synthesized following the procedure described by Peters *et al.* (18). Methane sulfonyl chloride (630 mg, 5.5 mmol) was added to a suspension of sodium hydroxide (240 mg, 6 mmol) in dichloromethane (5 ml) at 0°C under a nitrogen atmosphere. (*E*)-pent-2-en-4-yn-1-ol (410 mg, 5 mmol) was added and the mixture was stirred overnight at room temperature. The reaction mixture was diluted with dichloromethane and basified with a saturated sodium hydrogencarbonate solution. The organic layer was dried, filtered and concentrated under reduced pressure at 30°C. The crude product was used directly without purification.

A mixture (1 ml) of the activated alcohol in a mixture of formic and acetic acid (1:1) was added to *S*-adenosyl-l-homocysteine (38.4 mg, 0.1 mmol) under nitrogen atmosphere. The reaction mixture was protected from light and stirred at room temperature for 30 h. The reaction was followed by liquid chromatography-mass spectrometry (LCMS) [isocratic at 0.1% solution of formic acid in milli-Q purified water (MQ)]. On completion, the reaction was quenched with MQ water (15 ml) and extracted with diethylether (3 × 30 ml). We found that this crude extract can be used directly in transalkylation reactions by the DNA methyltransferases (M.TaqI, M.XbaI and M.FokI). However, to know product concentration and purity, we further purified the AdoEnYn. In this case, the aqueous layer was lyophilized and purification was performed by preparative reverse-phase high pressure (or high performance) liquid chromatography (isocratic at 0.1% solution of formic acid in MQ, retention time = 5.12 min.).

Imaging was performed using an Olympus IX71 microscope coupled to a red, 100 mW, 641 nm, solid-state diode laser (Spectra Physics). Fluorescence emission was recorded via a 700/75 nm band-pass filter (Chroma), using a Hamamatsu Image-EM camera. Data analysis was performed using ‘Localizer’, a freely available plug-in for Igor-Pro for super-resolution image analysis (25). Subsequent alignment and comparative analysis of the data were performed in Matlab. Images of the maps were generated using Circos (26).

## RESULTS

### Screening for DNA methyltransferase activity with the AdoEnYn cofactor

We screened 11 wild-type methyltransferase enzymes (taken from all three classes of methylases: cytosine-C5, cytosine-N4 and adenine-N6) for activity in the presence of the AdoEnYn cofactor (Figure 1). This cofactor carries a transferable 2-penten-4-yn group, an alkyne-terminating chain, that is covalently bound to either an adenine or cytosine (depending on the methyltransferase specificity) by the methyltransferase. Of the enzymes we screened, we found that only three showed significant activity with AdoEnYn and that all of these enzymes target the N6-amino moiety of adenine for methylation/alkylation. Along with M.TaqI (target sequence 5′-TCG**A**-3′ (the underlined base is the target for methylation), M.XbaI (target sequence 5′-TCTAG**A**-3′) and M.FokI (full length M.FokI targets both 5′-GG**A**TG-3′ and 5′-C**A**TCC-3′) methylases displayed efficient transalkylation of DNA with reactions typically reaching completion in 2–4 h. The wild-type methyltransferases that showed little or no activity with the AdoEnYn cofactor were M.HhaI, M.EcoRI, M.EcoDam, M.PvuII, M.BsaHI, M.PstI, M.HpaII and M.SssI (see Supplementary Figure S3).

It was previously found (27) that the activity of the C5-cytosine methyltransferases can be significantly enhanced by introducing up to three mutations in the region of their cofactor-binding pocket. The conserved nature of the amino acid sequences for this group of enzymes allows us to make, for example, analogous mutations to the M.BsaHI enzyme. We did so and tested the enzymatic activity using the same restriction assay as we applied to the wild-type enzymes. While we noted some decrease in the activity of the mutant M.BsaHI enzymes with the AdoMet cofactor, they failed to display any significant activity with the AdoEnYn cofactor (Supplementary Figure S4).

### Damage to DNA during the CuAAC

**Figure 2.**
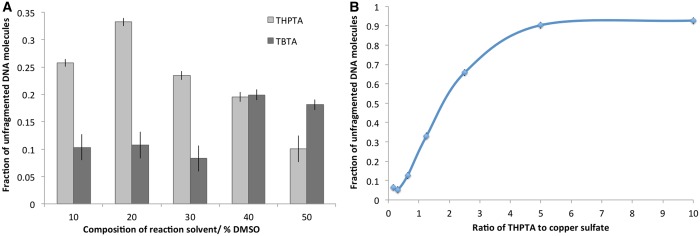
Plots derived from the agarose gel-based analysis (Supplementary Data) showing the extent of DNA damage incurred as a result of the CuAAC reaction under different reaction conditions. Plots show the amount of protected pUC19 plasmid DNA, calculated from the integrated intensity of the supercoiled and nicked bands (‘undamaged’ DNA) and compared with the integrated intensity of the smear resulting from fragmented DNA molecules in the gel. (**A**) Histograms showing the extent of DNA damage incurred during the CuAAC reaction in the presence of the THPTA or TBTA ligands, as a function of solvent composition. (**B**) Plot showing the effect of increasing the THPTA concentration, relative to the copper sulphate concentration in the CuAAC reaction on DNA damage.

We used the CuAAC (28) to fluorescently label the DNA at the alkylated sites. It is has been shown that the presence of a copper-coordinating ligand, such as the triazole TBTA (29) can be used to both accelerate the reaction and prevent DNA damage (30), at least on oligos and small PCR-generated DNA fragments. We tested the application of TBTA and the more hydrophilic ligand THPTA over a range of mixed solvent (aqueous buffer:DMSO) conditions to establish the optimal composition for protection of the DNA from Cu-mediated cleavage during the CuAAC reaction. Figure 2 shows a histogram derived from an agarose gel (Supplementary Figure S1) of pUC19 DNA molecules that have been incubated for 1 h in the presence of copper sulphate, ascorbic acid and either one of two stabilizing ligands (TBTA or THPTA). This mixture generates and stabilizes the Cu(I) used as the catalyst in the CuAAC reaction.

Figure 2A shows that, despite the presence of a copper-coordinating ligand, at a 1:1 ligand:copper ratio, the majority of the large DNA molecules in the sample are damaged during the CuAAC reaction. The composition of the solvent for this reaction does plays a role in facilitating the protection of DNA and the THPTA ligand is more effective in preventing DNA damage than TBTA.

Unlike TBTA, where the ratio of the ligand to copper must be maintained around 1:1 to obtain good azide-alkyne coupling efficiencies (31), the concentration of THPTA, relative to copper, can reportedly be as high as 10:1 without detrimentally impacting on the coupling reaction efficiency (30). Hence, we also examined the effect of using an excess of THPTA on the prevention of DNA damage. Figure 2B shows that an excess of 5 - to 10-fold THPTA is sufficient to prevent any significant breakdown of the plasmid DNA molecules, though the majority of the DNA is in the nicked open circular conformation.

**Figure 3.**
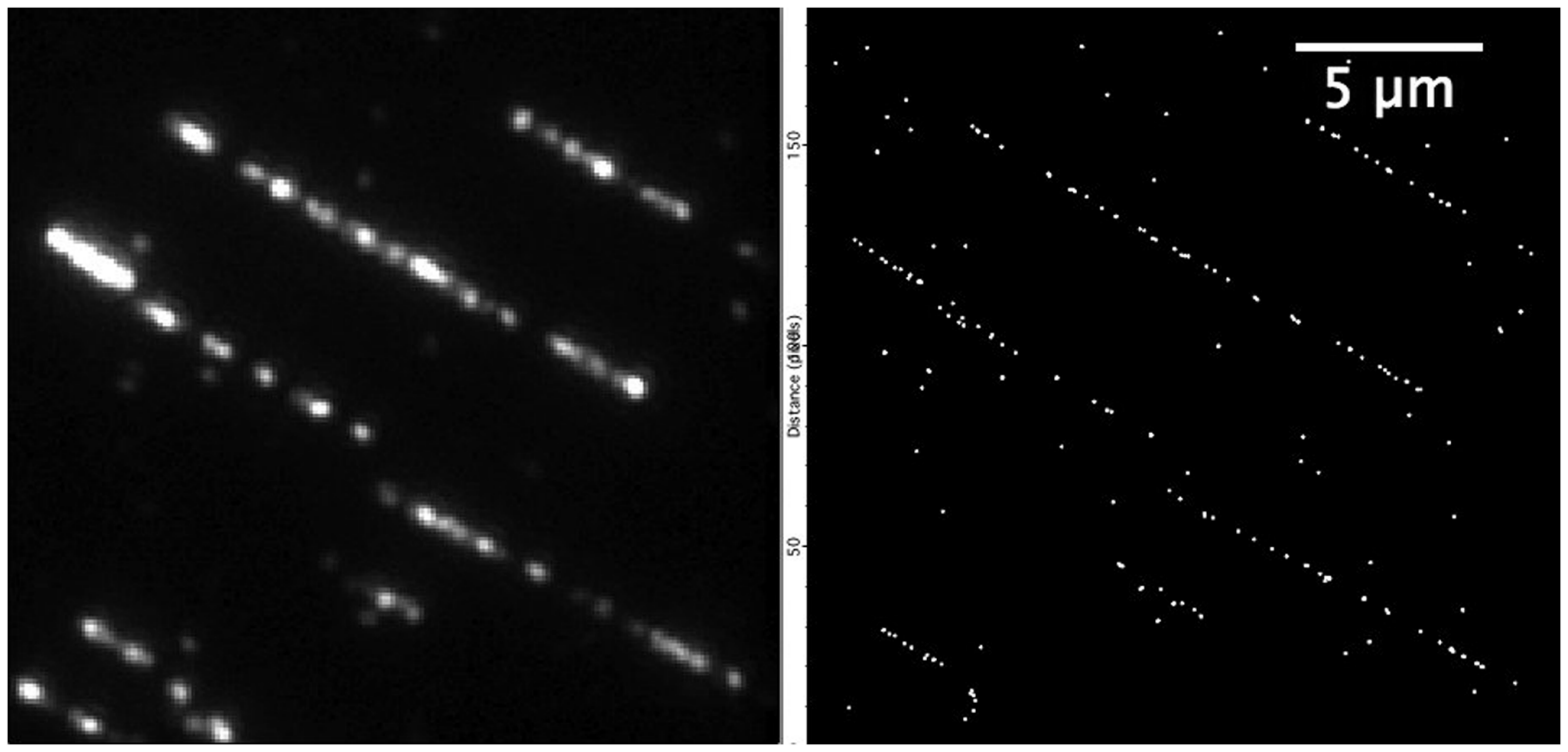
Standard wide-field (left frame) and super-resolution (right frame) images of labelled (TaqI-directed) T7 DNA linearized on a glass surface using molecular combing.

We prepared fresh DNA samples, labelled under conditions optimized to minimize DNA damage for both TBTA and THPTA ligands and mounted those on coverslides using molecular combing to examine the efficiency of labelling under these conditions. An image of a typical field-of-view of the microscope is depicted in Figure 3 (more examples can be found in Supplementary Figure S2). As expected from the agarose gel, the DNA is relatively fragmented, despite the stabilizing ligands, though this situation appears to be improved for the THPTA sample. Furthermore, we found that, for example, if the reaction mixtures were stored at 4°C overnight fragmentation continued, which is a clear indication that the fragmentation is due to copper-mediated cleavage of the DNA and not to other experimental factors such as DNA shearing as the result of handling.

### DNA deposition via molecular combing

Following purification of the DNA, it is deposited onto a glass cover slide, which is modified using an alkyl-silane such that it has a hydrophobic surface suitable for molecular combing. We achieve DNA deposition by slowly translating a droplet containing < 1 ng of DNA over this surface. Molecular combing of the DNA results in its near-uniform stretching to 1.59 ± 0.03 times its crystallographic length, where the standard deviation is calculated based on the 14 measurements reported for the M.TaqI/TBTA mapping. Hence, for a molecule of 10 kb in length, the variability in stretching typically gives rise to a standard deviation in the distance measurement of just <200 bp.

### Single-molecule genomic DNA mapping

The three methylases that display activity with the AdoEnYn cofactor target 111 sites (M.TaqI), 97 sites (M.FokI) or just three sites (M.XbaI) on the T7 bacteriophage genome. We prepared fluorocode maps of the T7 genome with all three enzymes and for M.TaqI in combination with the labelling using both the TBTA and THPTA ligands. In the case of the M.TaqI and M.FokI enzymes, this necessitates recording a movie of the individual DNA molecules, such that the emission from individual fluorophore labels can be identified and localized with super-resolution precision (typically within 10 nm, ∼20 bp). We implemented this using the ‘bleaching analysis’ in Localizer (25), a freely available analysis package for super-resolution microscopy. An example of the raw experimental data and the derived super-resolution image is shown in Figure 3. The M.XbaI enzyme targets sites that are well separated when the DNA is combed onto a surface and imaged using standard wide-field microscopy. Hence, the fluorescence emission from individual fluorophores is well separated in space and their locations can be determined (also in Localizer) from a single image. However, to trace the DNA molecule between these fluorophores we also stain the DNA with an intercalating dye (YOYO-1). In such a way, it is possible to derive a highly accurate, yet low-density, map of the DNA from just two images (red/M.XbaI-directed labels and green/intercalating dye). Unfortunately, the extent of the DNA cleavage that we observe, coupled with the imperfect labelling efficiency makes low-density mapping using M.XbaI extremely challenging. Images showing typical fields-of-view for each of the mapping projects and the derived super-resolution images are shown in Supplementary Figure S2.

**Figure 4.**
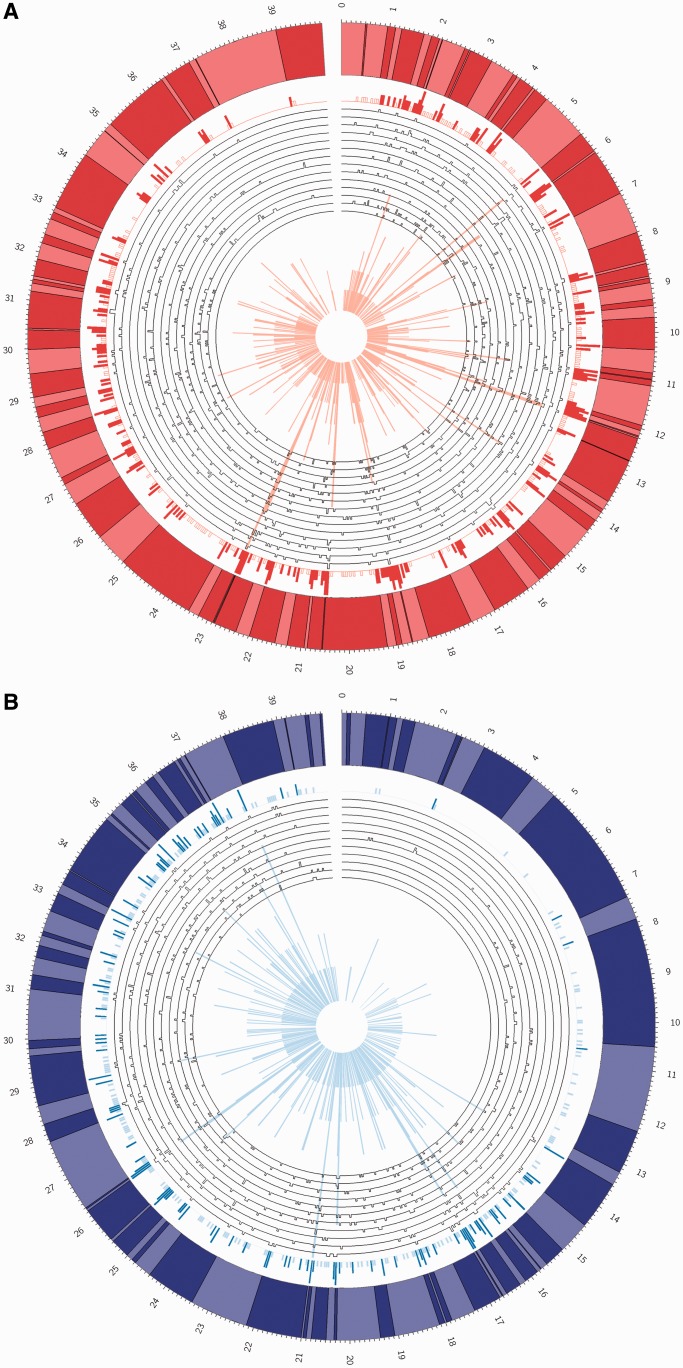
Circular histograms generated from individual DNA molecules and compared with the ‘reference’, *in silico*-generated map (outer plot in which every line going from light to dark indicates a site) for the given methyltransferase on the T7 phage genome. Histogram (**A**) shows the fluorocode map derived from the genome treated with M.TaqI enzyme. Histogram (**B**) is the M.Fok-derived fluorocode map. Data from the individual DNA molecules is shown in the uncoloured plots, with each peak indicating the position of a localized fluorophore. These data are then gathered together in the outermost histogram. For clarity, this outermost histogram is scaled from 0 to ≥5 for the M.TaqI map and 0 to ≥4 for the M.FokI map. The darker peaks are used to emphasize those sites containing multiple fluorophores. The innermost histogram also shows the plot of all the experimental data together in a single histogram but with the full range of the ‘y’-axis. Counts go from 0 to 10 for the M.TaqI-derived histogram and from 0 to 6 for the M.FokI-derived plot.

Following the image analysis, individual DNA molecules are selected and the positions of labelling sites on each molecule are compared with an *in silico*-generated reference map for a given enzyme of the T7 genome. The molecules are aligned by translating one relative to the other over a range of stretching values, applied uniformly to the experimental data set and typically limited to between 0.6 and 0.65 times the measured length. The optimal overlap of the two plots is determined and each experimentally derived molecule is assigned a ‘shift’ and ‘stretch’ factor that allows direct translation of the measured data points into locations on the genome (i.e. a translation of the distance in nanometres into a distance in base pairs). Finally, the experimental data are plotted in a histogram to give a plot of counts (target sites) per bin along the genome. Each bin of the histogram has a width of 50 bp and these histograms are plotted (M.TaqI and M.FokI) circularly in Figure 4 (linear versions of these plots can be found in Supplementary Figure S7 along the raw data in an Excel spreadsheet).

## DISCUSSION

### A subset of methyltransferases perform DNA transalkylation with AdoEnYn

Eight of the 11 methyltransferases we screened showed no apparent activity with the AdoEnYn cofactor. Images of the agarose gels used in screening reactions are shown in the Supplementary Figure S3A–G. Recently published work (27,32) shows that single point mutations around the cofactor binding pocket of a methyltransferase can have a dramatic effect on the activity of a methyltransferase enzyme for a given cofactor. All of the three cytosine-C5 DNA methyltransferases modified in this way were shown to display enhanced activity with some (though not all) synthetic AdoMet analogues. We made a series of mutants of the M.BsaHI enzyme, containing one, two or all three of the Q82A, Y254S and N304A mutations. These mutations are derived from those identified by Lukinavičius *et al.* for the M.HhaI enzyme and are readily determined as a result of the conservation of specific amino acid motifs across the cytosine C5 methyltransferases. Despite the promise of this enzymatic engineering, we found no significant rate enhancement for the transalkylation reaction with these mutants of M.BasHI and the AdoEnYn cofactor (Supplementary Figure S4). Our results suggest that while the engineering of the cytosine-C5 methyltransferases may enhance their activity with some cofactors this cannot reliably be extended to encompass all possible cofactor/enzyme combinations.

To maximize the chance of finding an active enzyme, many of the enzymes we selected for this study bear some relationship to M.TaqI, which has shown activity with a broad range of AdoMet analogues. The most closely related (in terms of amino acid sequence identity) is M.PstI, which derives from the same subclass of methyltransferases as M.TaqI (Adenine N6, type IIγ) (11). Despite this, we observe different activity of these enzymes with the AdoEnYn cofactor. Sequence alignment (33) and comparison of the amino acid sequences (Supplementary Figure S4) of M.TaqI and M.PstI shows that, while there is good alignment of known conserved motifs of M.PstI and M.TaqI, the M.PstI sequence contains four significant insertions (∼50 more amino acids) relative to the M.TaqI methylase on the N-terminal side of the conserved catalytic ‘NPPY’ motif; i.e. in the cofactor-binding region of the enzyme.

We modelled the M.PstI structure using the Phyre2 (34) server and comparison with the experimentally derived M.TaqI structure (PDB ID: 1G38) suggests that at least one of these insertions lies in close proximity to the cofactor (Supplementary Figure S6). In fact, the largest insertion of sequence, in M.PstI relative to M.TaqI, occurs at the N-terminus of the enzyme, which in the modelled structure sits directly adjacent to the cofactor. Unfortunately, this region is also highly flexible and so is not resolved in the M.TaqI structure (or the M.PstI model). While the effect of this structural difference appears clear from our experiments, there remains a significant amount unknown about the specific interactions that prevent these enzymes from forming catalytically competent complexes with the emerging generation of synthetic cofactors.

### DNA damage under optimal conditions for the CuAAC reaction

The CuAAC reaction has previously been shown to result in complete or near-complete labelling of alkyne-modified nucleotides in short DNA molecules. El-Sagheer and Brown (28) have, for example, reviewed the remarkable efficiency of Huisgen’s cycloaddition reaction for labelling DNA, where the conjugation efficiency of azides to alkyne-modified DNA has been shown to exceed 95%. Hence, from this perspective, and combined with its high specificity, the CuAAC is ideally suited to the labelling of DNA for optical mapping. To achieve maximal reaction rates and coupling efficiencies, the reaction requires careful optimization and this has been tested rigorously and reported recently (31,35). Hence, our aim was, on one hand, to use these reaction conditions to achieve effective DNA labelling, while on the other hand, balancing this with our need to maintain long genomic DNA molecules throughout the reaction (36–38).

We know of no report of CuAAC for labelling large genomic-scale DNA molecules (although labelling of chromatin *in vivo* has been demonstrated) (39). Our results show that, even for the relatively short plasmid molecule used in tests (Figure 2), while DNA damage can certainly be significantly reduced by the use of a Cu(I)-coordinating ligand, each lane of the gel (Supplementary Figure S1) displays a significant smear of randomly fragmented DNA. Figure 2 shows that between ∼10 and 30% of the DNA is unharmed (or nicked) under the CuAAC reaction conditions in 1 h. Each ligand has an optimal solvent mixture for ensuring protection of the DNA; 40% DMSO for TBTA (∼20% protected) and 20% DMSO for the THPTA ligand (∼33% protected). This result is surprising because these two well-known ligands have been shown to stabilize the (transiently formed) Cu(I) catalyst for the CuAAC reaction in solution and minimize the extent of DNA damage in labelling reactions containing PCR fragments of a similar size to the plasmid DNA we use (40). We also examined the effect of varying the relative amount of THPTA to Cu in the reaction mixture for negation of DNA damage. THTPA is thought to bind copper less tightly than TBTA and, hence, it can be used in several-fold excess in the reaction mixture without significantly retarding the reaction rate. We found that a 10-fold excess of THPTA to copper sulphate can be used to prevent the majority of DNA damage during the click reaction.

We applied both ligands under the conditions that we determined as optimal for protection DNA rationalizing that the negative effects of any random DNA fragmentation should be offset by an expected labelling efficiency for the CuAAC reaction as high as 95% and map densities on the sub-kilobase level. In the future, however, the solution to this problem is clearly to avoid the use of Cu(I) in the DNA labelling reaction. This can be done, for example, by using the strain-promoted copper-free azide-alkyne cycloaddition. This has recently been successfully demonstrated in the bulk phase by Lukinavičius *et al.* (41) The other ‘click’ series reactions (such as the Diels-Alder cycloaddition, thiol-ene click chemistry, oxime formation) also offer promising options, though these options require significant further synthesis, optimization of labelling and purification and enzymatic screening.

### Single-molecule genomic mapping

We embarked on this study with the ultimate aim of deriving a reliable, super-resolution, genome-wide map from a single, or few, fluorescently labelled DNA molecules. Such a demanding application necessitates the optimization of multiple critical factors including labelling efficiency, labelling fidelity, protection of long DNA molecules, localization accuracy and the purification strategy.

As a result of the supreme labelling efficiency of the methyltransferase enzymes (readily detected using a restriction enzyme and gel electrophoresis), the overall labelling efficiency in the two-step mTAG labelling approach is determined by the efficiency of the second, fluorophore-coupling, step. Our previous work used amino-NHS ester (N-hydroxysuccinimidyl ester) coupling for this step and we found this resulted in rather low labelling efficiency of 34%, on average, which appears comparable with recently reported efficiency for this chemistry in the literature (42), though greater labelling efficiencies (∼85%) have been demonstrated (43). Regardless of the overall labelling efficiency, amine-NHS ester coupling suffers from several known drawbacks including a lack of specificity (proteins or other aliphatic amino-containing compounds present in the reaction mixture are likely to also be labelled), inherent instability of the NHS-ester towards hydrolysis and low (yet significant in this application) reactivity of the NHS-ester towards aromatic amino groups. Hence, here we felt it worthwhile to pursue a labelling approach based on the azide-alkyne cycloaddition reaction, which promises completely specific labelling with ∼100% efficiency. Ultimately, we found this to be a rather optimistic expectation but nevertheless we see a significant improvement in the current results versus our earlier study (12). Figure 4 shows the DNA fluorocode maps that are derived from multiple DNA fragments, where labelling was targeted with either M.TaqI (Figure 4A) or M.FokI (Figure 4B). If we consider the M.TaqI data, they are derived from images of molecules that were selected as being the longest available on the sample, which range from ∼23 to 36 kb in length, with an average length of ∼30 kb, three quarters of the length of the complete T7 phage genome. Each carries an average of 60 fluorophores, so ∼70% of the TaqI sites are labelled. Hence, while DNA damage, as a result of the CuAAC reaction, is undeniable, the fluorophore coupling efficiency is remarkably high when the complex nature of the labelling reaction (and in particular the nanomolar alkyne concentration) is taken into consideration. Table 1 gives an overview of the physical properties of the molecules we used for the fluorocode map construction. In optical restriction mapping, the typical digestion rate is ∼75%, comparable with our labelling efficiency. However, the occurrence of a ‘false positive’ site is far lower in restriction mapping; ∼2% of restriction sites are deemed ‘false positives’, compared with around a third of the labels we localize (44). This can be the result of many factors but is a rather arbitrary figure that is derived from our strict definition of a ‘false positive’ (>50 bases from a reference site).

**Table 1.** Quantitative analysis of the molecules used in fluorocode mapping

Methyltransferase	Ligand	Total number localized fluorophores	Average size of DNA molecule (kb)	Average number of fluorophores per molecule	Percent matched data	Percent matched reference sites
M.TaqI	TBTA	820	30	60	67	98.7
	THPTA	982	28	80	53	98.3
M.FokI	TBTA	471	25	44	52	93.0

The term ‘data’ refers to the experimentally derived fluorocode maps, whereas the ‘reference’ is the *in silico*-generated fluorocode map based on the known DNA sequence for the T7 bacteriophage genome. A ‘match’ between the ‘data’ and ‘reference’ is designated when a point (fluorophore) in the ‘data’ lies within 50 bp, i.e. in the same bin or an adjacent bin to a point on the reference map.

Despite the THPTA ligand providing better protection of the DNA during the CuAAC reaction, we found the TBTA reaction mixture purified more readily for our imaging experiments (as summarized in Table 1 and also in the Supplementary Figures S2, S7 and S8). Hence, we focus on data derived from the CuAAC reaction facilitated by the TBTA ligand here.

The M.FokI-directed labelling experiments resulted in a similar distribution of DNA molecule sizes as the M.TaqI experiments, with maps from 11 molecules varying from 25 to 30 kb in length. Labelling efficiency also appears similar with on average, 44 fluorophores mapped per DNA molecule, again, ∼70% of the available number sites.

A small proportion of the localized fluorophores in both maps cannot be matched to a site on the reference map in any given molecule. There are several possible sources of error in the experiment, for example, stretching that is non-uniform (including breaking of the DNA), non-covalent binding of fluorophores to the DNA duplex, surface-bound fluorophores that are coincident with a deposited DNA molecule or non-specific DNA modification by the methyltransferase enzyme. Control experiments using a sample with no covalent coupling of fluorophore to DNA (no sodium ascorbate added to the CuAAC reaction mixture) show that almost no Atto647N is co-deposited with the DNA following purification. This strongly suggests that at least some non-specific labelling is due to methyltransferase-directed mislabelling of sites.

Recent results from sequencing experiments have shown that some DNA methyltransferases are remarkably specific in their targeting of sites, while others are less so (45,46). Interestingly, the M.FokI enzyme has been well-studied in this regard, since it is composed of two domains that have distinct recognition sequences (GGATG/CATCC). Friedrich *et al.* (47) characterized the star activity (the ability of the enzyme to modify sites that differ from the known target sequence by one base) of the two M.FokI domains independently and found that, while the N-terminal domain (recognizing GGATG) is rather specific, the C-terminal domain will also readily modify sites that differ by one base from its CATCC recognition sequence, with a rate reduction in the methylation reaction of only 1- to 3-fold. We see some indications from the maps we produced that M.FokI may retain less specificity than M.TaqI, for example, in Figure 4B there are several clusters of sites in the experimental map that do not correspond to expected sites for FokI labelling in the reference map. For example, there is one such cluster at ∼34.3 kb. This region, however, does correspond with a known star site (AGATG/CATCT) for both the N- and C-terminal M.FokI methylases and its labelling is certainly consistent with the expected star activity of the M.FokI enzyme. We see no such clusters (hundreds of bases away from the known sites for the enzyme) in the M.TaqI map, implying that this error is an enzymatic ‘mistake’, due to the star activity of M.FokI, though, due to the low probability of star activity, we would need to collect far more data to confirm this.

In conclusion, we present a super-resolution map of DNA sequence with increased specificity and carrying a greater information density compared with any previous map. Such an increase in labelling efficiency and specificity is absolutely critical to the application of methyltransferase-directed labelling for optical DNA mapping. We have shown that the high labelling efficiency, derived from the CuAAC reaction, combined with precise fluorophore localization using super-resolution imaging means that the DNA sequence can be studied at high map density (one site every few hundred bases) on a single molecule basis. We also show that the length of long genomic DNA molecules can be preserved during the CuAAC reaction by using a 10-fold excess of THPTA and that this does not significantly impact on labelling efficiency, though the non-specific association of fluorophores with the DNA duplex following this reaction is troublesome. Our maps were prepared using a cofactor derived from a two-step synthesis, a commercially available dye (Atto647N-azide) and commercially available methyltransferase enzyme (M.TaqI) to define the labelling sites. Furthermore, imaging data were analysed using an open-source super-resolution software package (Localizer) (25). Future work will focus on developing an approach to avoid DNA damage during labelling, which will allow a long-range and readily prepared map of much larger genomes than described here. With such improvements we envisage the methyltransferase-based mapping approach finding important applications in genomics and particularly in *de novo* sequence assembly and in studying unculturable, or rapidly evolving populations of organisms or for single-cell genomics experiments (48).

## SUPPLEMENTARY DATA

Supplementary Data are available at NAR Online.

## FUNDING

Personal fellowship from ‘Instituut voor Wetenschappen en Technologie’ [IWT110562 to R.K.N.]; European Research Council under the European Union's Seventh Framework Programme [FP7/2007-2013]/ERC Grant Agreement [291593 FLUOROCODE], the Flemish government in the form of long-term structural funding ‘Methusalem’ [METH/08/04 CASAS], the ‘Fonds voor Wetenschappelijk Onderzoek Vlaanderen’ [FWO grants G.0197.11; G0484.12] and the Hercules Foundation [HER/08/021 to J.H.]. Funding for open access charge: European Research Council, Grant Agreement n° 291593 FLUOROCODE.

*Conflict of interest statement*. R.K.N., V.L. and J.H. are founding members of an incubator-stage spin-off, Chromometrics, that aims to provide a DNA mapping service using the DNA methyltransferase-directed labelling described in the manuscript.

## Supplementary Material

Supplementary Data
