# Supplementary material for: Super-resolution optical DNA Mapping via DNA methyltransferase-directed click chemistry
Source: Nucleic Acids Res. 2014 Jan 21;42(7):e50. doi: 10.1093/nar/gkt1406 (PMC3985630; doi:10.1093/nar/gkt1406)
Supplement: Supplementary Data [file supp_42_7_e50__index.html]

Super-resolution optical DNA Mapping via DNA methyltransferase-directed click chemistry — Super-resolution optical DNA Mapping via DNA methyltransferase-directed click chemistry — Supplementary Data 

# Super-resolution optical DNA Mapping via DNA methyltransferase-directed click chemistry

## Supplementary Data

files

**Files in this Data Supplement:**

- Supplementary Data - docx file
- Supplementary Data - xlsx file
