# Supplementary material for: Super-resolution optical DNA Mapping via DNA methyltransferase-directed click chemistry
Source: Nucleic Acids Res. 2014 Jan 21;42(7):e50. doi: 10.1093/nar/gkt1406 (PMC3985630; doi:10.1093/nar/gkt1406)
Supplement: Supplementary Data [file supp_gkt1406_nar-03074-met-g-2013-File006.docx]

**SUPPLEMENTARY DATA**





Figure 1. Agarose gel showing the influence of ligands and variation in DMSO:buffer ratio in the CuAAC reaction for stained and labelled pUC19 plasmid DNA. The gels (Lonza Flash Gel) show stained pUC19 plasmid DNA following incubation under conditions used to generate the copper(I) catalyst in the CuAAC reaction. A) The catalyst was coordinated using either TBTA and THPTA, as indicated. The lanes for each ligand are reactions carried out in (from right to left) 10%, 20%, 30%, 40% and 50% DMSO by volume in the reaction mixtures. B) DNA damage as a result of a series of two-fold dilutions of the THPTA ligand. THPTA concentration is 2mM, 1mM, 0.5mM, 0.25mM, 0.125mM, 63µM, 32µM and 16µM, fro right to left. Copper sulfate concentration was 200µM in all lanes. The ladder is the Lonza Flash Gel 100-4000bp ladder.


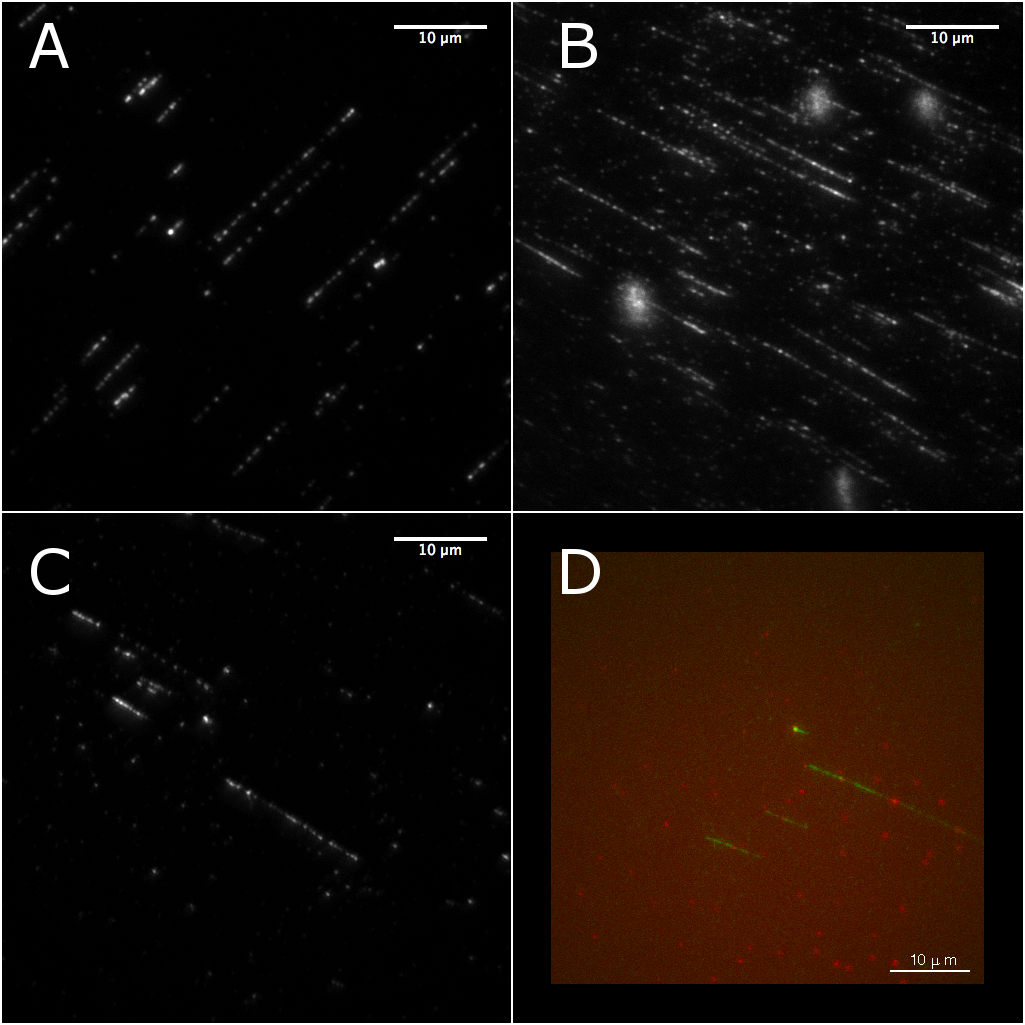


Figure 2. Images showing typical fields-of-view from the DNA mapping experiments. A: M.TaqI-directed labeling of the T7 genome using TBTA as the Cu(I)-coordinating ligand for the CuAAC reaction. B: M.TaqI-directed labeling of the T7 genome using THPTA as the Cu(I)-coordinating ligand for the CuAAC reaction. C: M.FokI-directed labeling of the T7 genome using TBTA as the Cu(I)-coordinating ligand for the CuAAC reaction. D: Two-colour image of the M.XbaI-directed labeling experiment. Intercalating YOYO-1 dye is shown in green, with the M.XbaI-directed labels shown in red.


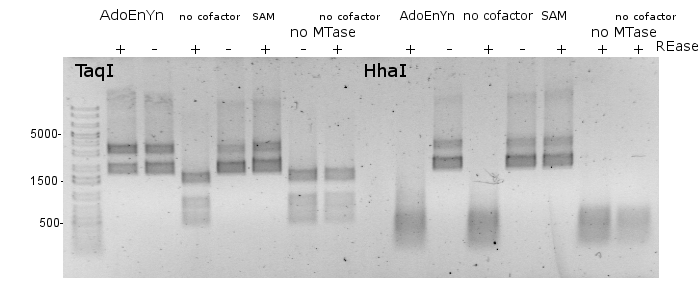


Figure 3A. Protection assay for M.TaqI and M.HhaI on pUC19 plasmid DNA. [M.TaqI]. M.TaqI shows complete protection of the plasmid DNA with the AdoEnYn cofactor in 2h. Restriction digests were performed using R.TaqI (M.TaqI) or R.HhaI (M.HhaI). Lane 1: 1kb plus DNA ladder (Fermentas). Lanes 2 and 3: assay with AdoEnYn cofactor and M.TaqI; lanes 4 and 5: control reactions with no cofactor added; lane 6: assay confirming M.TaqI activity with native cofactor, SAM; lane 7: control without methyltransferase added; lane 8: control digest without methyltransferase and without cofactor added. [M.HhaI] M.HhaI displays no observable activity with the AdoEnYn cofactor in 2h. Lanes 1 and 2 assay with AdoEnYn cofactor and M.HhaI; lanes 3 and 4: control reactions with no cofactor added; lane 5: assay confirming M.HhaI activity with native cofactor, SAM; lane 6: control reaction without methyltransferase added; lane 7: control reaction without methyltransferase and without cofactor added.


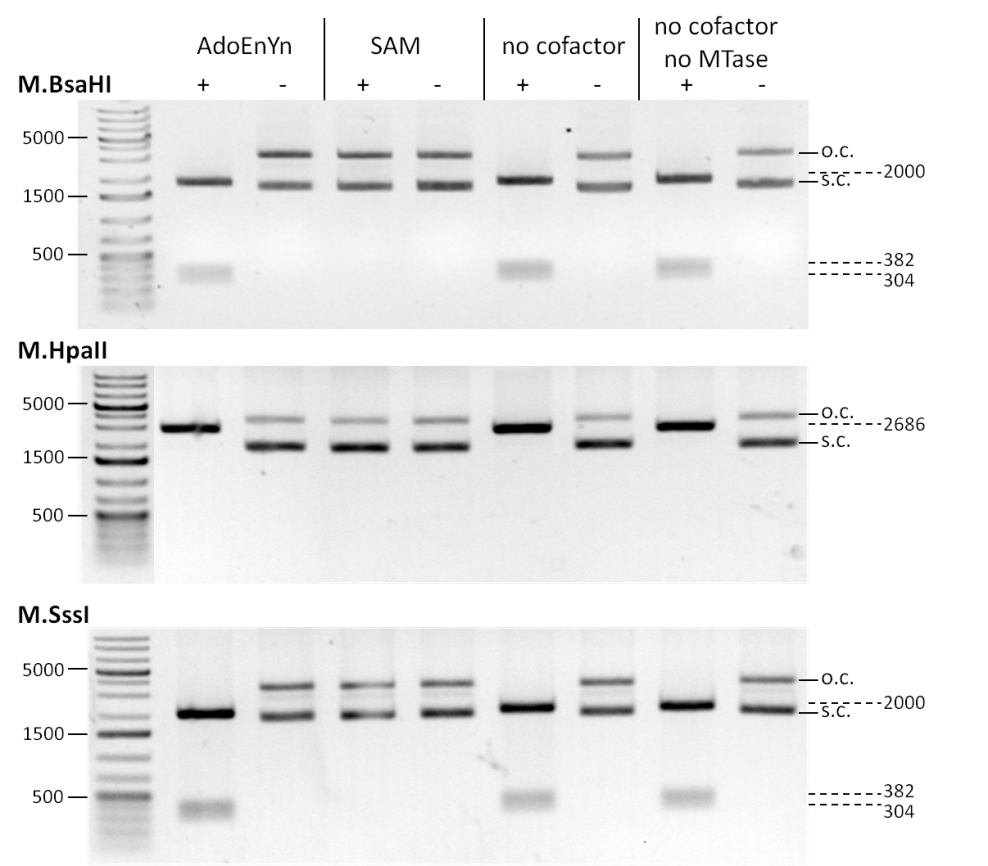


Figure 3B. Protection assay with three m5C methyltransferases on pUC19 plasmid. Lane 1: 1kb plus DNA ladder (Fermentas). Lanes 2-9 show different reaction conditions for the methylase, followed by either ‘+’, a challenge of the DNA by a restriction enzyme, or ‘-‘, no challenge. The restriction enzyme is selected such that it cleaves DNA only in the absence of methylation/alkylation. Lanes 2 and 3: assay with AdoEnYn; lanes 4 and 5: control assay with SAM; lanes 6 and 7: control assay without a cofactor; lanes 8 and 9: control assay without a cofactor and MTase. Lane 2 contains fully digested DNA for all three methyltransferases, hence the three enzymes are unable to use AdoEnYn as cofactor. To the right of the gels, the full lines indicate intact, undigested DNA; dotted lines indicate fragments of digested DNA; fragment size is indicated in bp; o.c.: open-circular plasmid; s.c.: supercoiled plasmid.


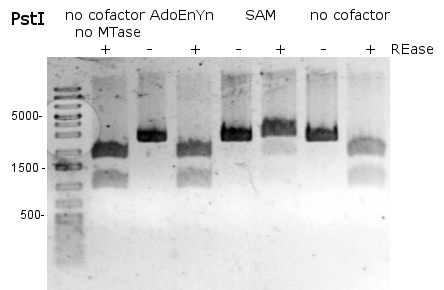


Figure 3C. Protection assay for M.PstI on R.SacI-linearized pUC19 plasmid DNA. Lane 1: 1kb plus DNA ladder (Fermentas). Lane 2: control assay showing digestion of the linearized DNA molecule with no protection; lanes 3 and 4: assay with AdoEnYn cofactor and PstI methylase; lanes 5 and 6 assay confirming M.PstI activity with native cofactor, SAM; lanes 7 and 8 control reactions with no cofactor added. M.PstI shows no apparent activity with the AdoEnYn cofactor.


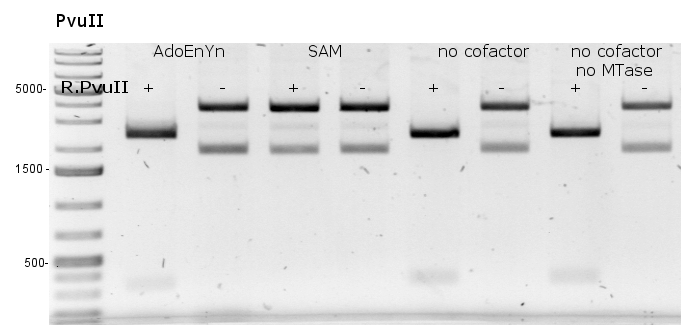


Figure 3D. Protection assay for M.PvuII on pUC19 plasmid DNA. DNA is digested in the assay with AdoEnYn, although a faint band of linear DNA is visible, protection appears very slow. Restriction digests were performed using R.PvuII. Lane 1: 1kb plus DNA ladder (Fermentas). Lanes 2 and 3: assay with AdoEnYn; lanes 4 and 5: control assay with SAM; lanes 6 and 7: control assay without a cofactor; lanes 8 and 9: control assay without a cofactor and methyltransferase.


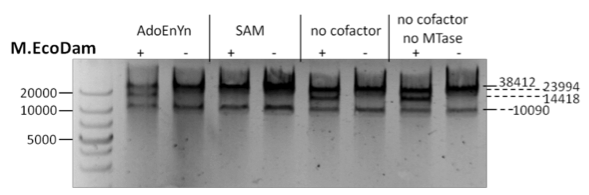


Figure 3E. Protection assay for M.EcoDam on (unmethylated) phage lambda DNA. The E.coli Dam methylase shows no observable activity with the AdoEnYn cofactor. Restriction digests were performed using R.ApaI and R.XbaI. Cleavage by R.XbaI is blocked by M.EcoDam methylation. Lane 1: 1kb plus DNA ladder (Fermentas). Lanes 2 and 3: assay with AdoEnYn cofactor and M.EcoDam; lanes 4 and 5: control reactions with SAM; lanes 6 and 7: control digest without cofactor added; lanes 8 and 9: control digest without methyltransferase and without cofactor added.


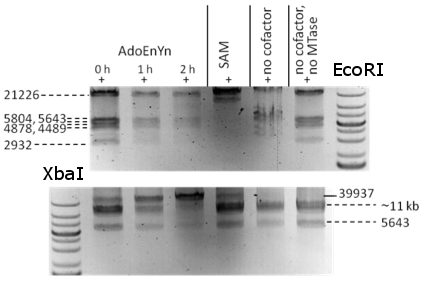


Figure 3F. Protection assays for M.EcoRI on phage lambda DNA and M.XbaI on T7 phage DNA. [M.EcoRI (Top)] M.EcoRI shows no observable activity with the AdoEnYn cofactor. Restriction digests were performed using R.EcoRI. Lanes 1-3: assay with AdoEnYn cofactor and M.EcoRI, reaction progress was followed over 2h; lane 4: control reaction with SAM; lane 5: control digest without cofactor added; lane 6: control digest without methyltransferase and without cofactor added; lane 7: 1kb plus DNA ladder (Fermentas). [M.XbaI (bottom)] M.XbaI gives almost complete protection of the T7 genome with the AdoEnYn cofactor in 2h. Lanes in the gel are as above with the ladder shifted to lane 1.


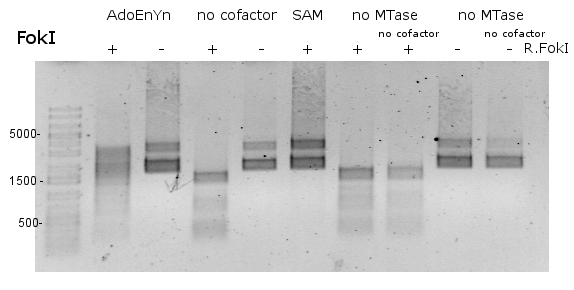


Figure 3G. Protection assay for M.FokI on pUC19 plasmid DNA. M.FokI shows near complete protection of the plasmid DNA with the AdoEnYn cofactor in 2h. Restriction digests were performed using R.FokI. Lane 1: 1kb plus DNA ladder (Fermentas). Lanes 2 and 3: assay with AdoEnYn cofactor and M.FokI; lanes 4 and 5: control reactions with no cofactor added; lane 6: assay confirming M.FokI activity with native cofactor, SAM; lane 7: control digest without methyltransferase added; lane 8: control digest without methyltransferase and without cofactor added; lane 9: control without methyltransferase added; lane 10: control without methyltransferase and without cofactor added.


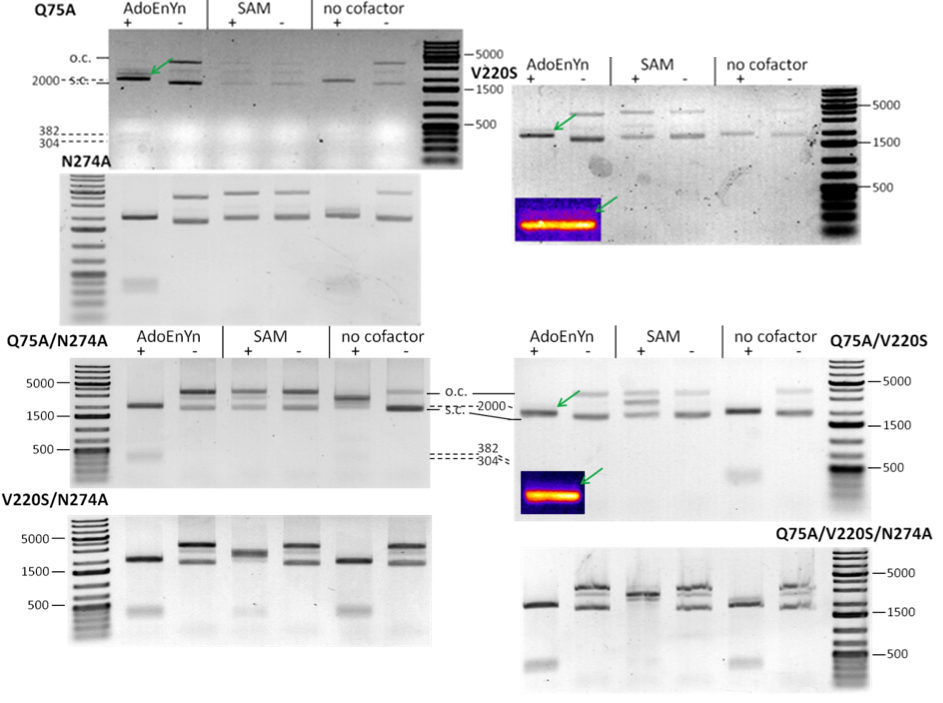


Figure 4. Protection assays for single, double and triple mutants of the M.BsaHI enzyme with the AdoEnYn cofactor. The mutants display little detectable activity on the pUC19 plasmid DNA with the AdoEnYn cofactor in 2h. Restriction digests were performed using R.BsaHI. Lane 1: 1kb plus DNA ladder (Fermentas). Lanes 2 and 3: assay with AdoEnYn cofactor and indicated M.BsaHI muatant; lanes 4 and 5: control reactions with native cofactor, SAM; lane 7 and 8: controls without cofactor added. Methylation was carried out in cell lysates, where residual AdoMet from the cell extract was depleted by the addition of an oligonucleotide containing the M.BsaHI recognition sequence, prior to the addition of the plasmid DNA and cofactor used in the screening. Green arrows and expanded regions show emerging bands in the gels that indicate a possible slow transalkylation reaction.

Figure 5. Alignment of M.TaqI and M.PstI sequences. The SAM-binding domain of M.TaqI lies to the N-terminal side of the highly-conserved (in adenine methyltransferases) catalytic motif ‘NPPY’, highlighted in red. The M.PstI enzyme contains significantly more amino acids than M.TaqI in this region, which may contribute to the lack of activity of M.PstI with the AdoEnYn cofactor. Regions highlighted in blue are those in the M.TaqI crystal structure where amino acids of the enzyme are within 5Å of the cofactor.


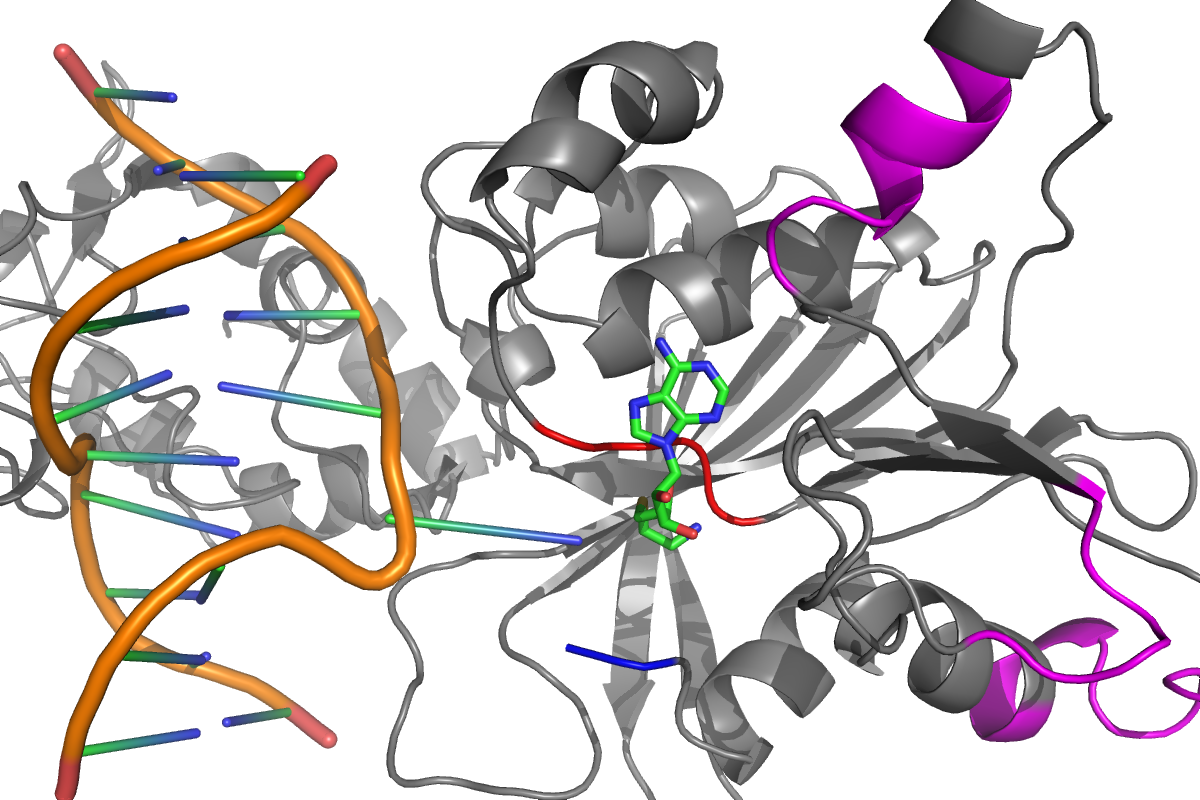


Figure 5. Model of the M.PstI structure (grey) generated using the Phyre2 server and by alignment to the M.TaqI crystal structure (pdb file 1G38). Also shown are the DNA (orange) and the cofactor analogue (5'-deoxy-5'-[2-(amino)ethylthio]adenosine) (green) from the M.TaqI crystal structure. The catalytic motif (NPPY) of the M.PstI is highlighted in red, the N-terminal in blue and the ‘inserts’ of sequence in M.PstI, relative to M.TaqI, in magenta.

Figure 6- Linear plots showing the frequency of counts in 50 base pair bins versus the genomic position for M.TaqI (with TBTA and THPTA coordinating compounds used as the ligand in the CuAAC labelling reaction) and M.FokI directed labeling (TBTA ligand) (black contours).Colored lines show the positions of sites for each enzyme on the bacteriophage T7 genome. These lines are arbitrarily scaled (y-axis) to aid visualization.


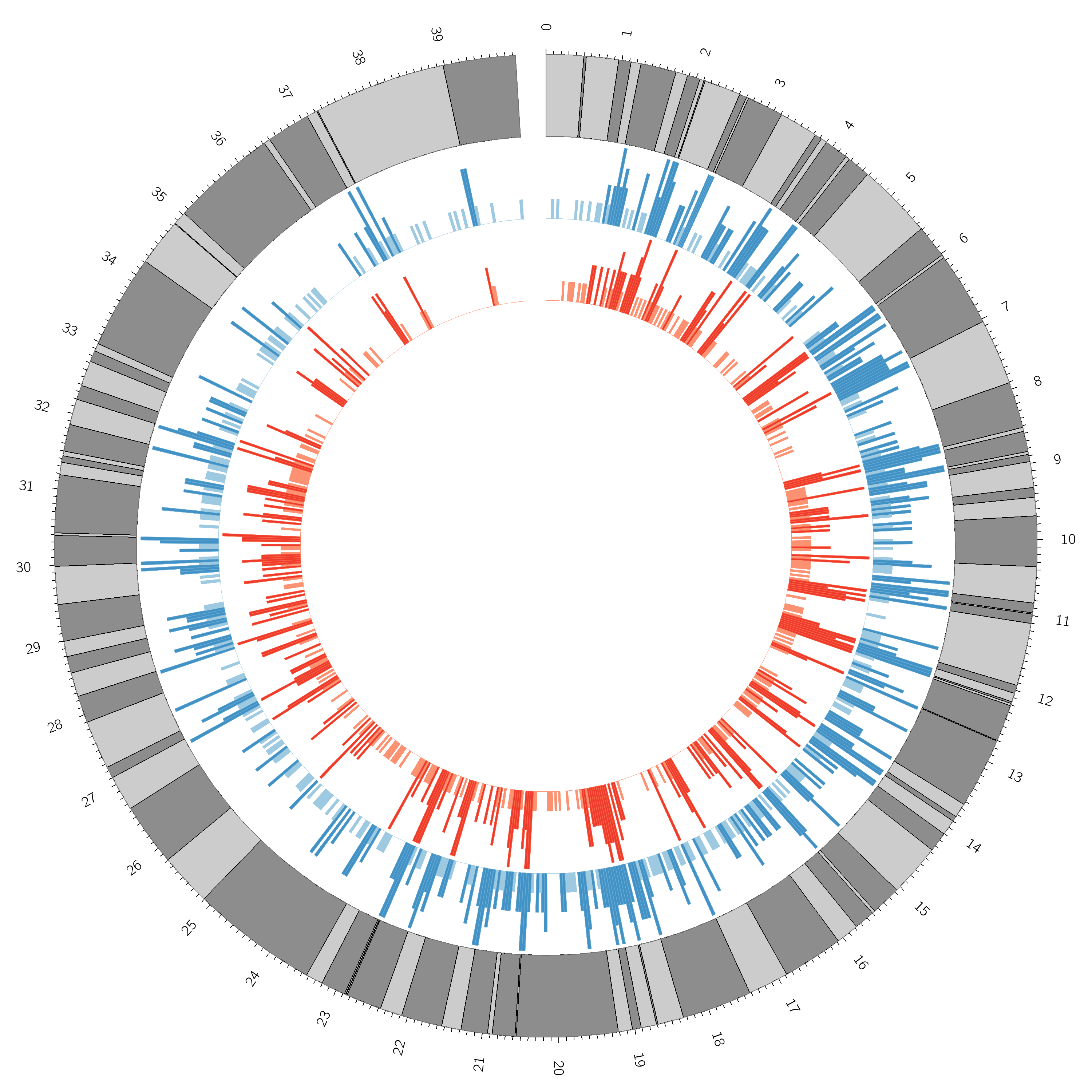


Figure 7- Circular plot of showing the locations (black lines) of the M.TaqI target sites on the 40 kb bacteriophage T7 genome (grey plot) and histograms of the experimental data from fluorocode mapping using etiher the TBTA (red) or THPTA (blue) ligand to facilitate the copper-catalyzed azide-alkyne cycloaddition reaction. Histogram bins are 50 bases in width and both plots are scaled from zero to four (or greater) counts. Note that the blue histogram (THPTA ligand) appears more noisy than the red (TBTA ligand) data. This is consistent with the images shown above in Figure 2.
